# Dose Responses to Supplemental Polyacrylamide on Digestion, Metabolism, and Ruminal Digestive-Enzyme Activities in Cattle

**DOI:** 10.3390/life15091487

**Published:** 2025-09-22

**Authors:** Yanqin Chen, Qiujiang Luo, Zhen Huang, Changjiang Zang, Rong Pan

**Affiliations:** 1 Laboratory of Animal Nutrition, College of Animal Science, Xinjiang Agricultural University, Urumqi 830052, China; xjdxcyq@sohu.com (Y.C.); huangzhen@163.com (Z.H.); zcj780@126.com (C.Z.); 2319117721@qq.com (R.P.); 2 College of Chemistry and Chemical Engineering, Xinjiang Agricultural University, Urumqi 830052, China

**Keywords:** PAM, intake, digestibility, rumen, cattle

## Abstract

In recent years, in response to the demand for the livestock industry to enhance cattle production performance, scholarly inquiries have centered on elucidating the underlying mechanisms by which feed additives modulate rumen microenvironment and metabolic efficacy, thereby facilitating nutrient absorption and augmenting production performance in cattle. This study was undertaken to evaluate the impacts of surfactant polyacrylamide (PAM) supplementation on digestive processes, metabolic dynamics, and ruminal digestive enzyme activities in cattle. Four ruminally cannulated crossbred cows (~3 years, 350 kg, non-pregnant/lactating) were utilized in a 4 × 4 Latin square design. The animals were fed a basal diet supplemented with polyacrylamide (PAM) at concentrations of 0, 1.0, 2.0, and 6.0 g/kg across four 22-day experimental periods, each consisting of a 16-day adaptation phase and a 6-day sampling phase. Supplementation with polyacrylamide (PAM) at levels ranging from 1.0 to 6.0 g/kg of diet significantly increased voluntary dry matter intake (VFI) in cattle by a maximum of 13.7% (*p* < 0.05), with peak effects at 2.0 g/kg. The digestibility of dry matter, crude protein, cellulose, and energy was significantly improved (*p* < 0.05 to *p* < 0.01), reaching maximum increases by 12.6%, 12.8%, 17.5%, and 11.7%, respectively. Nitrogen, calcium, and phosphorus retention increased substantially (*p* < 0.01 to *p* < 0.05), with calcium retention showing the highest improvement (55.7%). Rumen cellulase activities (endocellulase, exocellulase, cellobiase, and xylanase) were significantly enhanced (*p* < 0.01), peaking at 37.3% for cellobiase. However, pectase, amylase, and protease activities remained unaffected. Optimal benefits were observed at 2.0 g/kg PAM, highlighting its potential to improve feed efficiency and nutrient utilization in cattle.

## 1. Introduction

Enhancing productive performance of ruminants has a valuable role in sustainable agricultural systems and the provision of food to human beings. However, ruminants play a pivotal role in converting vast renewable resources derived from rangeland, pasture, and crop residues, or other by-products, into edible food, with the assistance of the rumen. The rumen of these animals houses a diverse and intricate microflora, encompassing bacteria, protozoa, archaea, and fungi. This microbial community collectively plays a vital role in the transformation of plant material into nutrients that can be utilized [1,2]. However, in practice, the digestion of concentrates by ruminants is not as efficient as that of mono-gastric farm animals. But previous studies have indicated that modulating rumen microbial populations and their metabolic processes can lead to improvements in animal productivity [3,4,5]. Enhancing production performance via the modulation of ruminal fermentation has long been a focal area of interest within ruminant nutrition research. Prior investigations have demonstrated that surfactants possess the capability to influence the rumen microbial ecosystem, alter ruminal fermentation dynamics, and ultimately boost animal productivity [6,7,8]. For instance, it has been documented that incorporating nonionic surfactant, alkyl polyglycoside (APG), into the diet can improve the utilization of fatty acids by rumen bacteria and alter the functional capabilities of goat bacteria [6]. Similarly, a separate study has indicated that adding APG to the diets of lactating cows results in a linear increase in the non-fat solid and total solid contents of milk [7]. Another investigation conducted on sheep revealed that introducing the nonionic surfactants Tween 60 and Tween 80 into their diet impacts rumen metabolites. This intervention leads to a decrease in ammonia nitrogen levels and an elevation in the concentration of volatile fatty acids, but voluntary feed intake (VFI) and digestion was unaffected [8]. An anionic surfactant known as docusate (also referred to as DOC or aerosol OT) has been found to enhance nutrient absorption in the small intestine [9]. Additionally, it has demonstrated the ability to increase nitrogen retention in sheep [10,11] and improve the slaughter performance of lambs [11]. The addition of DOC (docusate) to cattle diets does more than just influence their feed intake and digestion; it also improves nitrogen retention in cattle [12] and boosts nutrient digestion and milk output in dairy cows [13]. Polyacrylamide (PAM), an anionic surfactant akin to docusate (DOC), is often employed as a chemical auxiliary in oil fields [14], as a clarifying agent in water treatment plants [15], or as a clarifying agent in fruit juice processing [16]. Supplementary PAM has been shown to enhance the digestive processes, metabolic efficiency, growth rates, and carcass qualities of lambs [17], while also boosting nutrient digestion and milk production in dairy cows [13].

Surfactants not only affect rumen metabolites but also exert an influence on rumen microflora. For example, the biosurfactant mannosylerythritol lipid has been observed to decrease the population of most Gram-positive bacteria in the rumen, reduce methane production, and elevate the propionate-to-acetate ratio [18]; DOC supplementation led to decreased protozoan count in the diet, increased total amount of bacteria in rumen [19], and increased activities of fiber-degrading enzymes in rumen fluid [20]. PAM supplementation also decreased protozoan count, increased total amount of bacteria in rumen, and increased activities of fiber-degrading enzymes in the rumen [21].

Interestingly, it was found that the manipulation of rumen metabolism and flora, or of digestion and metabolism of the whole body by a surfactant or other chemicals, was dose responsible [12,19,21,22]; the dosages which increase voluntary feed intake (VFI) were usually suitable for improving the nutrition of animals.

For instance, when PAM was supplemented at doses of 1.0, 2.0, 3.0, and 6.0 g/kg of diet in sheep, it led to an increase in voluntary feed intake (VFI) by 8.8%, 17.9%, 8.8%, and −5.5%, respectively, and activities of fiber-digestive enzymes in rumen by 7.1%, 17.9%, 10.8%, and −1.5%, respectively, [21]; and when PAM was supplemented at a dosage of 2.0 g/kg of diet, the VFI, daily body weight, nitrogen retention, and carcass weight increased by 14.4%, 15.2%, 35.4%, and 24.5%, respectively, [17].

But there is still a lack of dose impact of PAM on cattle. Therefore, in the present study, the effects of PAM supplementation by different doses on VFI, digestion, metabolism, and ruminal activities of digestive enzymes were examined in cattle.

## 2. Materials and Methods

### 2.1. Experimental Design and Animals

The use of animals in this study was approved by the Animal Care Committee, Xinjiang Agricultural University (Approval No. 31772625, 5 April 2016, Urumqi, Xinjiang, China). Furthermore, the experimental protocols employed adhered strictly to the university’s established guidelines for animal research.

Four Simmental × Xinjiang Brown F1 non-pregnant female cattle, aged about 2.5 years, with a body weight of about 350 kg, were fitted with a permanent rumen fistula. Animals were individually fed with the same diet ad libitum. PAM was supplemented at doses of 0 (the control), 1.0, 2.0, and 6.0 g/kg dietary dry matter (DM) to determine the dose responses of supplemental PAM on digestion, metabolism, and ruminal digestion and metabolism.

The experiment diets were administered to animals in 4 × 4 Latin square design (*n* = 4 for each treatment). Each experimental period lasted for 22 days, with 16 days designated for acclimation to the new PAM dose and 6 days reserved for sample collection. Four experimental trials were conducted from August to November 2016, with the dates for each trial as follows: 5 August to 26 August (Trial 1), 29 August to 16 September (Trial 2), 19 September to 10 October (Trial 3), and 13 October to 3 November (Trial 4). In each experimental period, samples of pure feces and a mixture of urine and feces were collected for a digestibility and metabolism trial, and samples of rumen fluid were collected for assaying ruminal flora (unpublished data) and activities of digestive enzymes.

### 2.2. Diet, Feeding, and Management

The dietary ingredients and nutrients composition are shown in Table 1. Cornstalk was chopped to 4~6 cm size using forage cutter (9Z-20, Wanying Machinery Equipment Co., Ltd., Zhengzhou, China) and offered to animals ad libitum. The amount of cornstalk was controlled to preserve the cornstalk: the concentrate ratio constant was at 60:40 throughout the experiment. Experimental animals were kept in individual pens and had free access to feed and water. The feeding frequency was twice a day and feed was offered to the animals at 09:00 and 19:00, respectively. The concentrate was fed first and, after being fully consumed, cornstalks were offered. PAM is evenly mixed into the concentrate and fed to cattle. The amount of the diet was adjusted according to the previous day’s oats to allow for 2% to 4% refusal.

The amounts of concentrate and cornstalk offered, and the cornstalk residue, were documented daily for calculations of VFI. The pens were cleaned daily and kept dry. The rumen cannula was examined and maintained regularly.

Following the end of each period, the rumen fluid from control group cattle was taken through the cannula and transferred into the rumen of the other cattle undergoing PAM treatment via their rumen cannula; this procedure was performed with 400 mL of each fluid twice a day for three days to recover the ruminal microflora.

### 2.3. Sample Collection, Pretreatment, and Storage

During the final 6 days in each period, feed samples, feces, mixture of feces and urine, and residual cornstalk were collected daily. Immediately before feeding, 600 g of the concentrate and 800 g of chopped cornstalk were collected daily and kept at 4 °C. At the period’s end, all samples for each animal were pooled, subsampled, air-dried, and ground through a 1 mm sieve by high-speed disintegrator (FW 200, Guowang Machinery Equipment Co., Ltd., Changzhou, China). Refused cornstalk from each animal was collected, kept at 4 °C, pooled, and subsampled at the end of the period. Subsequently, these samples were used for the analysis of their chemical compositions.

The concrete floor of the animal house was thoroughly cleaned in the morning of day 17 of each period to collect mixed fecal and urine samples (urine-contaminated feces) for six days. Interval spot fecal samples without urine contamination, each approximately 250 g, were collected at six-hour intervals for six days, then stored at 4 °C. At the end of the period, samples from each cattle were pooled, subsampled, and air-dried. At 10:00 and 20:00 each day, all of the fecal and urine mixtures (feces and urine could not be separated) for each animal were collected, weighed, and mixed, and 5% of the mixture (by weight) was taken as a sample. The sample was mixed with 1 mL of 50% HCl to decrease the pH. In total, 12 mixture samples were collected for each animal. The samples were air-dried and ground through a 1 mm sieve for chemical analyses.

Rumen fluid samples, with an approximate 60 mL each, were collected on a daily basis through the cannula at specific time points, namely 0 h (prior to feeding), and 1.5, 3, 5, 7, and 10 h following the morning feeding, over the final three days of each experimental period. Consequently, each animal yielded triplicate samples at every designated time point. The rumen fluid was subjected to filtration through a 40-mesh double-layer nylon bag, and the nylon bag was gently squeezed. Subsequently, the filtered rumen fluid was thoroughly mixed, and two 5.0 mL aliquots were carefully withdrawn, and a drop of saturated mercuric chloride was added.

### 2.4. Chemical Analyses

The samples of feed, refused feed, feces, and urine were analyzed using the following methods described by AOAC [23]: dry matter (DM) (Code 930.15), organic matter (OM) (Code 942.05), crude protein (CP) (Code 990.03), calcium (Ca) (Code 920.39), and phosphorus (P) (Code 946.06).

The concentrations of neutral detergent fiber (NDF), acid detergent fiber (ADF), and acid detergent lignin (ADL) in both feed and fecal samples were precisely quantified by employing the standardized methodologies outlined by Van Soest et al. [24]. The levels of hemicellulose (HC), cellulose (CEL), and ADL were calculated as the differences between NDF and ADF levels before and after 72% H_2_SO_4_ treatment of ADF and after combustion of H2SO4-treated residue, respectively.

### 2.5. Digestive Enzyme Activities of Rumen Fluid

The enzymatic activities of endocellulase, exocellulase, exocellulase, xylanase, pectinase, amylase, and protease within the rumen fluid were precisely measured using standardized and validated analytical techniques.

The activity of endocellulase was precisely determined, employing 0.5% sodium carboxymethyl cellulose (CMC-Na) solution as the substrate [25]. Briefly, 0.5 g of sodium carboxymethyl cellulose (CMC-Na) was thoroughly dissolved in 100 mL of a 50 mM phosphate-buffered solution with a pH of 6.0. Subsequently, 1 mL of the resulting substrate solution was accurately pipetted into a 10 mL graduated test tube. The test tube was then placed in a 39 °C water bath and subjected to continuous oscillation for a duration of 30 min. Then, 1 mL of warmed rumen fluid was added to the reaction system and incubated for exactly 3 min, and 2 mL of dinitrosalicylic acid (DNS) was immediately added to stop the reaction. Subsequently, the sample was placed in a boiling water bath for a duration of precisely 10 min, followed with immediate cooling by running water. Sample was brought up to an exact volume of 10 mL using distilled water, shaken, and centrifuged at 1500× *g* for 10 min. The supernatant was used for the assay at OD550 [26].

The substrate employed for determination of exocellulase activity was a 0.5% (*w*/*v*) solution of microcrystalline cellulose [25]. The determination procedure adhered to the same protocol as that employed for assessing endocellulase activity. But the volume of the substrate and the diluted rumen fluid utilized in this assay were 1 mL and 0.6 mL, respectively.

The substrates utilized for the assessment of cellobiose [27], xylanase [25], and pectinase [28] activities were 0.5% (*w*/*v*) solutions of salicin, xylan, and pectin, respectively. The determination protocols were consistent with those described for the endocellulase activity assay, and, for all experimental groups, the volume of the substrate utilized was uniformly set at 1 mL. But the diluted rumen fluids were 1 mL, 0.5 μL, and 0.5 μL, respectively, [27].

The substrate for the assessment of amylase activity consisted of 0.5 mL of a 1% (*w*/*v*) starch solution, which was combined with 60 μL of rumen fluid. After incubation for 3 min at 39 °C, the enzymatic reaction was stopped by the addition of 1.0 mL of 3,5-dinitrosalicylic acid (DNS) reagent. Subsequently, the reaction mixture was immersed in boiling water for an additional 10 min duration. Upon cooling the mixture under running water, the volume was adjusted to 100 mL. Finally, the value at OD540 was determined [29].

For determination of the protease activity, 1.0 mL of rumen fluid and 1.0 mL of 2% casein were preheated in 39 °C water bath for 2 min, respectively, and incubated together for another 3 min. After an addition of 2 mL of 0.4 M trichloroacetic acid and incubation in water bath at 39 °C for 20 min, the samples were centrifuged at 1800× *g* for 10 min. A total of 1.0 mL of the supernatant was added to a reaction vessel, followed by the sequential addition of 5.0 mL of a 0.4 M sodium carbonate solution and 1.0 mL of a diluted Folin reagent. After each addition, the contents were thoroughly mixed to ensure uniform distribution of the reactants. The values at OD660 were determined after incubation at 39 °C for 20 min [30].

The procedures for constructing the standard curves for various enzyme activities were the same as described for sample determination. The glucose was used for the standard curves of the activities for endocellulase, exocellulase, and cellobiose ranges; xylose for the xylanase activity, galacturonic acid for the pectinase activity, maltose for the amylase activity, and tyrosine for the protease activity, respectively.

### 2.6. Calculations

The formulas for the calculation of feed intake; nutrient levels in pure feces and in the mixture of feces and urine; apparent digestibility and retention of nitrogen (N), Ca, and P were as follows [12]:

(1) Digested nutrient (kg/day) = Nutrient intake (kg/d) − Spot feces (kg/d) × [ADL intake (kg/d) ÷ ADL in spot feces (kg/d)];
(2) Apparent digestibility of nutrient (%) = Digested nutrient (kg/d) ÷ Nutrient intake (kg/d) × 100%;
(3) N, Ca or P in urine (kg/d) = Feces and urine mixture weight (kg) + Spot feces weight (kg) − Spot feces weight (kg) × ADL intake (kg/d) ÷ ADL in spot feces (kg/d);
(4) Retention of N, Ca or P (g/d) = Intake (g/d) − Excretion in urine (g/d) − Spot feces (g/d) × ADL intake (kg/d) ÷ ADL in spot feces (kg/d).

### 2.7. Statistical Analysis

The effect of PAM supplementation on the parameters was analyzed in a model with a 4 × 4 Latin square design where animal, period, and treatment were considered factors. The model was as follows:

Yijk = μ + αi + βj + γk + εijk,

where Yijk is an observation, μ is the overall mean, α is the fixed effect of PAM (j = 1–4), β is the random effect of animal (i = 1–4), γ is the fixed effect of treatment period (k = 1–4), and εijk is the residual error. Unless specified otherwise, the variations in these means are discussed in the context of notable temporal (or time-related) trends. Multiple comparisons of treatment means were conducted using Duncan’s new multiple range test. Polynomial contrasts were used to determine the linear and quadratic effects of PAM supplementation. The analysis was performed using SPSS 17.0 software. The data are presented as the mean and standard error of the mean (SEM), and use the Tukey method to compare the means, with rumen enzyme activity being analyzed at different sampling time points after feeding. Statistical significance was declared with *p* values ≤ 0.05 (significant) or ≤ 0.01 (highly significant).

## 3. Results

### 3.1. Dose–Response of Supplementary PAM on Voluntary Feed Intake and Digestion of Cattle

The results of VFI are shown in Table 2. Compared with the control group, supplementation with PAM at doses of 1.0, 2.0, and 6.0 g/kg diet increased the dry matter (DM) VFI of cattle by 5.1%, 13.7%, and 2.2% (all above *p* < 0.05), respectively. The patterns of change in OM, concentrate, and roughage intake were consistent with that of DM intake, all showing a significant fit to a quadratic curve. Additionally, the results indicated that the highest intake occurred at the PAM supplementation level of 2.0 g/kg diet.

As shown in Table 3, PAM supplementation at 1.0, 2.0, and 6.0 g/kg of diet increased the digestibility of DM (8.4%, 12.6%, and 9.5%), OM (7.1%, 10.8%, and 8.5%), CP (7.5%, 12.8%, and 4.2%), cellulose (12.2%, 17.5%, and 10.3%) and energy (8.6%, 11.7%, and 9.8%) (all above *p* < 0.01 vs. control group) in cattle. The digestibility of hemicellulose, Ca and P, showed similar changes. These results indicated that dietary supplementation with PAM enhanced the apparent digestibility of nutrients in cattle diets, and the dose–response relationship of PAM supplementation exhibited a well-fitted quadratic pattern, with the optimal effect observed at the supplementation level of 2.0 g/kg.

Table 3 also showed the digested amounts of diet under different PAM supplementation doses. The digested amount of all nutrients was increased by PAM supplementation. The dose–response relationship for PAM supplementation followed a quadratic trend, with 2.0 g/kg identified as the optimal dose, resulting in significant increases in 28.0% (DM), 30.5% (CP), and 20.6% (energy) compared to the control group (all *p* < 0.05).

### 3.2. Dose–Response of Supplementary PAM on Nitrogen, Ca, and P Metabolism of Cattle

As shown in Table 4, supplementation of PAM at 1.0, 2.0, and 6.0 g/kg diet increased nitrogen retention of cattle by 23.8% (*p* < 0.01), 36.9% (*p* < 0.01), and 4.2% (*p* < 0.05), respectively, and the nitrogen retention rate increased by 16.3% (*p* < 0.01), 19.1% (*p* < 0.01), and 3.1% (*p* < 0.05), respectively. These results indicate that PAM supplementation improved nitrogen metabolism in cattle, with the optimal dosage determined to be 2.0 g/kg of diet.

The effect of PAM supplementation on the metabolism Ca and P metabolism was similar to that of nitrogen. The intake and urine-discharged Ca and P were all quadratically increased with PAM supplementation, peaking at 2.0 g/kg of PAM dosage. And at 2.0 g/kg of PAM dosage, Ca and P retention increased by 55.7% and 37.1% (both *p* < 0.05) compared with the control, respectively.

### 3.3. Dose–Response of Supplementary PAM on Activities of Digestive Enzymes in Rumen of Cattle

The influence of PAM on ruminal fluid enzyme activities is shown in Table 5. Results of the effect of PAM on enzyme activity in rumen fluid showed that, at three levels of PAM supplementation, the activities of cellulase in rumen fluid increased and were quadratically dose responsible, being 19.5%, 32.52%, and 25.2% (all above *p* < 0.01) for endocellulase, 16.4%, 32.8%, and 28.4% (all above *p* < 0.01) for exocellulase, 19.3%, 37.3%, and 31.9% (all above *p* < 0.01) for cellobiase, and 23.1%, 32.1%, and 16.7% (all above *p* < 0.01) for xylanase, respectively. The highest valves of all enzyme activity were observed at 2.0 g of PAM/kg diet. However, the activities of pectinase, amylase, and protease were not significantly affected by PAM supplementation.

## 4. Discussion

Surfactant acts as a catalyst to promote the enzymatic hydrolysis of lignocellulose, thereby facilitating the production of bioethanol and biobutanol [31,32]. Generally, it has been documented that nonionic surfactants, such as polyethylene glycol and Tween, can reduce the interaction between enzymes and lignin, and exert a positive influence on fiber degradation. It has also been reported that cationic surfactants exhibit an inhibitory effect on cellulase activity, consequently, exerting a negative impact on the enzymatic hydrolysis of cellulose [33]. However, the influence of anionic surfactants on cellulose degradation remains a matter of inconsistent conclusions in the existing research. Research has demonstrated that certain anionic surfactants, such as sodium dodecyl sulfate (SDS), can enhance cellulose conversion and delignification processes [34,35], whereas small-molecule anionic surfactants exhibit an inhibitory effect on enzymatic hydrolysis [31]. The biochemical mechanism governing bioenergy production from lignocellulose shares substantial similarities with that of rumen fermentation [36]. However, studies have shown that an appropriate amount of anionic surfactant in vivo can reduce the abundance of rumen ciliate protozoa, decrease ruminal pH and ammonia nitrogen levels, and enhance in vitro gas production and microbial protein synthesis, thereby exerting a positive effect on rumen fermentation [37,38]. Furthermore, the addition of anionic surfactants can exert a synergistic effect when combined with *Lactobacillus plantarum*, *Pediococcus acidilactici*, and *Enterococcus faecium*, thereby enhancing the fermentation characteristics and accelerating the degradation rate of neutral detergent fiber (NDF) in barley silage [39]. Supplementation of the anionic surfactant in the diet of ruminants may have positive impact on nutrient intake, digestibility, growth, and carcass parameters. Incorporating anionic surfactants into the diets of ruminants may have positive impact on nutrient intake, digestibility, growth performance, and carcass quality metrics. For instance, a previous study demonstrated that the incorporation of surfactants improved the in vitro digestibility of DM and OM in low-quality roughage [40]. Similarly, another in vitro experiment investigated the effect of treating barley silage with the anionic surfactant sodium dodecyl sulfate (SDS), and the results revealed an improvement in the degradability of neutral detergent fiber (NDF) in the silage [39]. Dietary supplementation with DOC, an anionic surfactant, at a dose of 0.8 g/kg diet has been reported to increase VFI by 23.6% [11] and 30.7% [9] in sheep, as well as enhance carcass weight by 22.6% [11]. Additionally, PAM, another anionic surfactant, has been shown to increase VFI of sheep, exhibiting effects consistent with those of other anionic surfactants reported in previous studies [9,11]. Supplementation of PAM in the diet of sheep at doses of 1.0, 2.0, 3.0, and 6.0 g/kg diet increased VFI by 8.8%, 17.9%, 8.2%, and 5.5% [21], respectively. A recent study has reported that the nitrogen retention, digestibility, carcass weight, and carcass lean weight increased by 30.3%, 14.4%, 24.5%, and 30.6%, respectively, at a PAM dose of 2.0 g/kg of diet [17]. In present study, compared with control, the maximum increases in VFI, DM digestibility, and nitrogen retention of cattle at a PAM dose of 2.0 g/kg of diet were 13.7%, 12.6%, and 36.9%, respectively, showing the active impact of PAM on cattle nutrition. The consistency of results observed in both cattle and sheep indicate that supplementation with PAM at an appropriate dosage can enhance feed intake in ruminant animals. Consistent with the findings in sheep, PAM supplementation significantly increased the activities of endocellulase, exocellulase, and cellobiase in cattle, while exerting no significant effect on the activities of pectinase, amylase, and protease. Interestingly, xylanase activity was not enhanced by PAM in sheep [21], and this trend was also observed in cattle in the present experiment. This phenomenon may be attributed to differences in ruminal microbial flora [21]. As shown in Table 6, the present study clearly demonstrates a positive linear correlation between cellulase activity in ruminal fluid and nitrogen retention (NR) in cattle. Specifically, the correlations between endocellulase, exocellulase, and cellobiase activities and NR were statistically significant. These findings suggest that an increase in ruminal cellulase activity contributes to the improvement of nutritional status in cattle.

Previous studies have demonstrated that dietary supplementation with deoxycholic acid (DOC) increases the digestible amount of diet and nitrogen retention in cattle, exhibiting a dose-dependent effect [12]. The present studies showed that polyacrylamide (PAM), another anionic surfactant, exerts a similar effect on the digestible amount of diet and nitrogen retention in cattle, and also exhibits a dose-dependent response. However, a discrepancy was observed in dietary digestibility: PAM supplementation increased digestibility, whereas DOC decreased it. This difference may be attributed to variations in the extent of VFI enhancement between the two treatments. Nevertheless, the observed improvement in digestibility with PAM supplementation is consistent with findings from previous studies, which demonstrated that appropriate dietary supplementation with PAM in dairy cows results in enhanced nutrient digestibility. [13]. Previously, it has been documented that supplementing dairy cows with the polymer gel pH20 increases body fat accumulation and prolongs milk production [41], potentially due to the physicochemical similarities between pH20 and PAM as gel-based substances.

PAM is a linear polymer, which can be completely dissolved in water. It has characteristics of hygroscopicity and good thermal stability and is used for flocculation, adhesion, resistance reduction, and thickening. PAM is widely used as a water purification agent [42,43,44], fruit juice clarifier [45], and adhesive in tablet formulation. It is also used in food and biomedical packaging [46], and to effectively stabilize the erosion of soil [47].

The properties of PAM are generally considered safe, non-toxic, and stable [48,49,50,51], but acrylamide (AA), as a free monomer in PAM products, is considered toxic [48]. According to the EU, the threshold of AA in food should be between 50 and 850 μg/kg; for example, 50 μg/kg for wheat-based bread, 350 μg/kg for biscuits and wafers, 500 μg/kg for French fries (ready-to-eat), 750 μg/kg for fried potato chips, and 800 μg/kg for ginger bread has been recommended [52].

In food-grade PAM, AA is less than 250 μg/kg. Consequently, in this experiment, the level of AA in the diet for 350 kg cattle was below 500 μg/kg, a concentration comparable to that present in ready-to-eat French fries. The European Food Safety Authority (EFSA) has established the benchmark dose level 10 (BMDL10) for acrylamide (AA) in humans at 170 μg/day per kilogram of body weight (BW) [53,54]. In this experiment, the daily intake of AA by cattle (with BW of 350 kg, 10 kg diet per day) was less than 8 μg/day/kg BW. Consequently, it is reasonable to hypothesize that the PAM levels in the feed were safe for the cattle, and the resulting beef would also be safe for human consumption.

## 5. Conclusions

The VFI, digestibility, and nitrogen retention in cattle exhibited were dose-responsive to PAM supplementation, with the maximum increases observed at a PAM dosage of 2.0 g/kg of diet by 13.7% for VFI, by 12.6% for DM digestibility, and by 36.9% for nitrogen retention. The effect of PAM supplementation was significantly associated with ruminal cellulase activities but not with other digestive enzymes, suggesting that the positive impact of PAM supplementation on cattle nutrition may be mediated by improvements in rumen function. However, the underlying mechanisms linking PAM-induced cellulase activation to improved nitrogen retention remain unclear, warranting further investigation into microbial community dynamics and metabolic pathways in the rumen. Additionally, current research is mostly short-term trials, and the sustained impacts of long-term PAM supplementation on rumen ecological balance and production performance remain unclear. To establish a theoretical foundation for the scientific application of PAM in livestock production, in the future, it is necessary to combine multi-omics technologies (metagenomics, metabolomics) to analyze the long-term mode of action of PAM and evaluate the dose–response differences under different feeding conditions (such as high roughage vs. high precision feed diets).

## Figures and Tables

**Table 1:** Dietary composition and chemical composition (DM basis).

Ingredients	%	Nutrient Levels ^b^	%
Corn stalk	59.26	Organic matter (OM)	91.84
Corn	24.73	Crude protein (CP)	14.03
Cottonseed meal	8.30	NDF	53.78
Rapeseed meal	2.79	ADF	30.98
Urea	0.72	ADL	3.39
Nutritive additive ^a^	4.20	Calcium (Ca)	0.77
		Phosphorus (P)	0.27
		Gross Energy (MJ/kg)	16.94

^a^ The nutritive additive provided the following nutrients per kilogram of the diet: NaCl 0.50%, Na_2_SO_4_ 0.42%, limestone 2.20%, Ca(HPO_4_)_2_ 0.80%, vitamin A 1350 IU, vitamin D 270 IU, vitamin E 45 IU, iron 16 mg, copper 8 mg, zinc 5 mg, manganese 10 mg, iodine 8.5 mg, cobalt 0.10 mg, and selenium 0.20 mg. ^b^ Nutrient levels were means of 3 measurements.

**Table 2:** The effect of dietary PAM supplementation on the VFI of Xinjiang brown × Simmental F1 cattle (kg/d, *n* = 4).

Item	Polyacrylamide g/kg of Diet				SEM	*p*-Value ^1^		
	0	1.0	2.0	6.0		Trt	Linear	Quadratic
DM	8.64 ^d^	9.08 ^b^	9.82 ^a^	8.83 ^c^	0.12	<0.001	0.277	<0.001
OM	7.73 ^d^	8.15 ^b^	8.87 ^a^	7.97 ^c^	0.11	<0.001	0.408	<0.001
Concentrates	3.57	3.76 ^b^	4.04 ^a^	3.64	0.49	<0.001	0.575	<0.001
Roughage	5.07 ^d^	5.33 ^a^	5.79 ^b^	5.19 ^c^	0.07	<0.001	0.757	<0.001

In the same row, the values of the same item with different small letter superscripts mean significant difference (*p* < 0.05). *p*-value ^1^: Trt means treatment effect of PAM; linear and quadratic effects of the PAM inclusion rate. DM means dry matter; OM means organic matter.

**Table 3:** The effect of dietary PAM supplementation on apparent digestibility of Xinjiang brown × Simmental F1 cattle (%, *n* = 4).

Item	Polyacrylamide g/kg of Diet				SEM	*p*-Value ^1^		
	0	1.0	2.0	6.0		Trt	Linear	Quadratic
Apparent digestibility (%):								
DM	56.7 ^d^	61.5 ^b^	63.9 ^a^	62.1 ^c^	0.74	<0.001	0.001	<0.001
OM	61.4 ^d^	65.7 ^b^	68.0 ^a^	66.6 ^c^	0.69	<0.001	<0.001	<0.001
CP	53.1 ^d^	57.1 ^c^	59.9 ^a^	55.3 ^b^	0.66	<0.001	0.342	<0.001
Cellulose	50.2 ^d^	52.1 ^c^	59.0 ^a^	55.4 ^b^	0.87	<0.001	<0.001	<0.001
Hemicellulose	57.2 ^d^	60.4 ^c^	65.4 ^a^	60.5 ^b^	0.76	<0.001	<0.001	<0.001
Energy	51.8 ^d^	56.3 ^c^	57.8 ^a^	56.9 ^b^	0.51	<0.001	0.002	<0.001
Ca	27.8 ^d^	34.9 ^b^	37.9 ^a^	33.2 ^c^	0.97	<0.001	<0.001	<0.001
P	28.8 ^d^	32.9 ^b^	35.8 ^a^	32.0 ^c^	0.65	<0.001	0.004	<0.001
Digested amounts (kg/day):								
DM	4.90 ^d^	5.58 ^b^	6.27 ^a^	5.48 ^c^	0.13	<0.001	0.002	<0.001
OM	4.75 ^d^	5.36 ^b^	6.04 ^a^	5.31 ^c^	0.12	<0.001	<0.001	<0.001
CP	0.59 ^d^	0.67 ^b^	0.77 ^a^	0.62 ^c^	0.02	<0.001	0.246	<0.001
Cellulose	1.15 ^d^	1.24 ^c^	1.54 ^a^	1.29 ^b^	0.04	<0.001	<0.001	<0.001
Hemicellulose	1.21 ^d^	1.34 ^b^	1.57 ^a^	1.31 ^c^	0.04	<0.001	0.015	<0.001
Energy (MJ/d)	83.9 ^d^	94.1 ^b^	101.2 ^a^	89.3 ^c^	1.79	<0.001	0.686	<0.001
Ca(g/d)	19.8 ^d^	26.1 ^b^	30.6 ^a^	23.4 ^c^	1.02	<0.001	0.051	<0.001
P(g/d)	7.42 ^c^	8.80 ^b^	10.2 ^a^	8.28 ^b^	0.26	<0.001	0.026	<0.001

In the same row, the values of the same item with different small letter superscripts mean significant difference (*p* < 0.05). *p*-value ^1^: Trt means treatment effect of PAM; linear and quadratic effects of the PAM inclusion rate. DM means dry matter; OM means organic matter; CP—crude protein; Ca—calcium; P—phosphorus.

**Table 4:** The effects of PAM supplementation on metabolism of N, Ca, and P in Xinjiang Brown × Simmental F1 cattle (g/cattle/day; *n* = 4).

Item	Polyacrylamide g/kg of Diet				SEM	*p*-Value ^1^		
	0	1.0	2.0	6.0		Trt	Linear	Quadratic
Nitrogen (g/d):								
Intake	177.9	189.2 ^b^	204.4 ^a^	179.6	2.76	<0.001	0.015	<0.001
In feces	83.4 ^b^	81.3	82.0	80.2 ^a^	0.47	0.091	0.033	0.490
In urine	52.5 ^b^	56.1	65.1 ^a^	55.7	1.33	<0.001	0.483	<0.001
Retention	41.9 ^d^	51.9 ^b^	57.4 ^a^	43.7 ^c^	1.65	<0.001	0.020	<0.001
Retention (%)	23.6 ^d^	27.4 ^b^	28.1 ^a^	24.3 ^c^	0.54	<0.001	0.173	<0.001
Calcium (g/d):								
Intake	71.2 ^d^	74.9 ^b^	80.7 ^a^	72.7 ^c^	0.95	<0.001	0.489	<0.001
In feces	51.5 ^a^	48.7 ^b^	50.1 ^b^	49.3 ^c^	0.30	<0.001	0.010	0.016
In urine	0.870 ^c^	0.970 ^b^	1.09 ^a^	0.91	0.02	<0.001	0.797	<0.001
Retention	18.9 ^d^	25.1 ^b^	29.5 ^a^	22.5 ^c^	1.00	<0.001	0.040	<0.001
Retention (%)	26.6 ^d^	33.6 ^c^	36.5 ^a^	30.9 ^b^	0.95	<0.001	0.002	<0.001
Phosphorus (g/d):								
Intake	25.8	26.7 ^b^	28.4 ^a^	25.9	0.27	<0.001	0.009	<0.001
In feces	18.3	17.9 ^a^	18.2	17.6 ^b^	0.09	<0.001	<0.001	0.720
In urine	0.730 ^d^	0.880 ^b^	0.980 ^a^	0.81 ^c^	0.03	<0.001	0.719	<0.001
Retention	6.69 ^d^	7.92 ^b^	9.17 ^a^	7.47 ^c^	0.23	<0.001	0.022	<0.001
Retention (%)	26.0 ^d^	29.6 ^c^	32.4 ^a^	28.9 ^b^	0.59	<0.001	0.001	<0.001

In the same row, the values of the same item with different small letter superscripts mean significant difference (*p* < 0.05). *p*-value ^1^: Trt means treatment effect of PAM.

**Table 5:** The effect of PAM on enzyme activity in rumen fluid of Xinjiang Brown × Simmental F1 cattle (IU(umol/min·mL), *n* = 4).

Item	Polyacrylamide g/kg of Diet				SEM	*p*-Value ^1^		
	0	1.0	2.0	6.0		Trt	Linear	Quadratic
Hours after feeding:								
Endocellulase:								
0	1.07 ^Cc^	1.38 ^Bb^	1.47 ^Aa^	1.39 ^Bb^	0.04	<0.001	<0.001	<0.001
1.5	1.19 ^Cc^	1.44 ^Bb^	1.55 ^Aa^	1.45 ^Bb^	0.04	<0.001	<0.001	<0.001
3	1.28 ^Dd^	1.51 ^Cc^	1.71 ^Aa^	1.58 ^Bb^	0.04	<0.001	<0.001	<0.001
5	1.36 ^Dd^	1.58 ^Cc^	1.79 ^Aa^	1.67 ^Bb^	0.04	<0.001	<0.001	<0.001
7	1.26 ^Dd^	1.47 ^Cc^	1.67 ^Aa^	1.60 ^Bb^	0.04	<0.001	<0.001	<0.001
10	1.20 ^Dd^	1.45 ^Cc^	1.59 ^Aa^	1.52 ^Bb^	0.04	<0.001	<0.001	<0.001
Average	1.23 ^Dd^	1.47 ^Cc^	1.63 ^Aa^	1.54 ^Bb^	0.04	<0.001	<0.001	<0.001
Exocellulase:								
0	0.57 ^Cd^	0.67 ^c^	0.76 ^Bb^	0.78 ^Aa^	0.03	0.002	0.001	0.007
1.5	0.63 ^Cc^	0.73	0.87 ^Aa^	0.82 ^Bb^	0.03	0.004	0.009	0.003
3	0.69 ^Cc^	0.78	1.01 ^Aa^	0.99 ^Bb^	0.04	0.001	0.002	0.007
5	0.78 ^Bb^	0.87	0.97 ^Aa^	0.96 ^Aa^	0.02	0.006	0.005	0.012
7	0.70 ^Cc^	0.84 ^Bb^	0.87 ^Aa^	0.83 ^b^	0.02	0.009	0.087	0.003
10	0.65 ^Bc^	0.77 ^b^	0.84 ^Aa^	0.77 ^b^	0.02	0.012	0.116	0.003
Average	0.67 ^Cd^	0.78 ^c^	0.89 ^Aa^	0.86 ^Bb^	0.03	0.002	0.003	0.002
Cellobiase:								
0	1.54 ^Dd^	1.78 ^Cc^	2.18 ^Aa^	2.07 ^Bb^	0.07	<0.001	<0.001	<0.001
1.5	1.60 ^Dd^	1.88 ^Cc^	2.30 ^Aa^	2.19 ^Bb^	0.07	<0.001	<0.001	<0.001
3	1.72 ^Dd^	2.00 ^Cc^	2.39 ^Aa^	2.31 ^Bb^	0.06	<0.001	<0.001	<0.001
5	1.82 ^Dd^	2.12 ^Cc^	2.32 ^Aa^	2.31 ^Aa^	0.06	<0.001	<0.001	<0.001
7	1.67 ^Dd^	2.10 ^Cc^	2.33 ^Aa^	2.17 ^Bb^	0.07	<0.001	<0.001	<0.001
10	1.61 ^Dd^	2.02 ^Cc^	2.16 ^Aa^	2.10 ^Bb^	0.06	<0.001	<0.001	<0.001
On average	1.66 ^Dd^	1.98 ^Cc^	2.28 ^Aa^	2.19 ^Bb^	0.06	<0.001	<0.001	<0.001
Xylanase:								
0	0.62 ^Cc^	0.83 ^Bb^	0.90 ^Aa^	0.83 ^Bb^	0.03	<0.001	0.011	<0.001
1.5	0.75 ^Dd^	0.94 ^Bb^	0.98 ^Aa^	0.90 ^Cc^	0.03	0.001	0.076	<0.001
3	0.85 ^Cc^	1.05 ^Bb^	1.15 ^Aa^	0.97	0.03	<0.001	0.305	<0.001
5	0.96 ^Bb^	1.04	1.15 ^Aa^	0.96	0.02	0.003	0.343	<0.001
7	0.81 ^Cc^	0.97 ^Bb^	1.02 ^Aa^	0.91	0.03	0.004	0.519	0.001
10	0.67 ^Dd^	0.90 ^Bb^	0.94 ^Aa^	0.87 ^Cc^	0.03	<0.001	0.003	<0.001
On average	0.78 ^Dd^	0.96 ^Bb^	1.03 ^Aa^	0.91 ^Cc^	0.03	<0.001	0.145	<0.001
Pectase:								
0	1.13	1.15	1.21	1.17	0.02	0.265	0.375	0.121
1.5	1.20	1.23	1.27	1.21	0.02	0.593	0.951	0.198
3	1.26	1.40 ^Aa^	1.38 ^b^	1.33	0.02	0.029	0.778	0.008
5	1.33	1.37	1.40	1.35	0.02	0.446	0.976	0.117
7	1.23	1.26	1.29	1.29	0.02	0.607	0.349	0.350
10	1.19	1.21	1.21	1.23	0.02	0.921	0.539	0.793
On average	1.22	1.27	1.30	1.26	0.02	0.480	0.657	0.145
Amylase:								
0	0.57	0.60	0.62	0.62	0.02	0.743	0.370	0.545
1.5	0.63	0.68	0.74 ^a^	0.68	0.02	0.114	0.458	0.026
3	0.72	0.77	0.81	0.73	0.02	0.246	0.755	0.053
5	0.78	0.78	0.79	0.78	0.02	0.999	0.916	0.938
7	0.69	0.71	0.70	0.71	0.02	0.941	0.707	0.883
10	0.61	0.67	0.65	0.65	0.02	0.731	0.703	0.458
On average	0.67	0.70	0.72	0.70	0.01	0.369	0.608	0.102
Protease:								
0	0.35	0.35	0.37	0.38	0.01	0.876	0.468	0.850
1.5	0.43	0.41	0.43	0.43	0.01	0.971	0.930	0.932
3	0.46	0.43	0.49	0.49	0.01	0.379	0.287	0.831
5	0.56	0.54	0.55	0.54	0.01	0.867	0.542	0.877
7	0.47	0.54	0.53	0.48	0.02	0.337	0.649	0.129
10	0.41	0.50	0.49	0.39	0.02	0.072	0.178	0.032
On average	0.45	0.47	0.48	0.45	0.01	0.190	0.676	0.039

In the same row, the values of the same item with different small letter superscripts mean significant difference (*p* < 0.05); and the different capital letter superscripts mean extremely significant difference (*p* < 0.01). *p*-value ^1^: Trt means treatment effect of PAM.

**Table 6:** Linear correlation between cellulase activity in rumen fluid and nitrogen retention (NR) in cattle.

Cellulase	*n*	Y(N g/Day) = a + bX (IU/mL)	R^2^	*p*-Value
Endocellulase	16	Y = 0.6401 + 0.017X	0.5135	<0.01
Exocellulase	16	Y = 0.3972 + 0.0083X	0.3696	<0.01
Cellobiase	16	Y = 0.8739 + 0.0237X	0.3864	<0.01
Xylanase	16	Y = 0.2732 + 0.0133X	0.8273	=0.145

## Data Availability

The original contributions presented in this study are included in the article. Further inquiries can be directed to the corresponding author.
